# A look through Latin America truck drivers’ health, a systematic review and meta-analysis

**DOI:** 10.1186/s12889-022-14902-2

**Published:** 2023-01-02

**Authors:** Silvia Veridiana Zamparoni Victorino, Fernanda Silva Oliveira, Vlaudimir Dias Marques, Constanza Pujals, Mariá Romanio Bitencourt, Ana Carolina Jacinto Alarcão, Thais Silva Santos, Mariana Teixeira da Silva, Fernando Castilho Pelloso, Wagner Sebastião Salvarani, Paulo Acácio Egger, Patrícia Costa Mincoff Barbanti, Lander dos Santos, Isaac Romani, Deise Helena Pelloso Borghesan, Daniel Augusto Message dos Santos, Sandra Marisa Pelloso, Raíssa Bocchi Pedroso, Maria Dalva de Barros Carvalho

**Affiliations:** 1 Postgraduate Program in Health Science, Health Science Center, Maringa State University, Avenida Colombo, 5790, Bloco 126. Jd. Universitário - CEP: 87020-900, Maringá, Paraná Brazil; 2 Regional University Hospital of Maringa, Maringa State University, Maringá, Paraná, Brasil; 3 Parana Adventist College, Ivatuba, Paraná Brasil; 4 Postgraduate Program in Bioscience and Patophysiology, Maringa State University, Maringá, Paraná Brasil; 5 Physician By the State Foundation for Health Care (FEAS), Curitiba, Paraná Brasil; 6 UNINGÁ- Ingá University Central, Maringá, Paraná Brasil

**Keywords:** Truck drivers, Chronic diseases, Meta-analysis, Systematic Review

## Abstract

Heavy truck drivers represent a social group of great importance to any country's economy. Their professional activity requires a high level of dedication. Due to the irregular hours in their work routine and adopted habits, they mostly predispose them to a diversity of health problems. The purpose of this study is to perform a systematic review and meta-analysis aiming to identify the prevalence of diabetes, hypertension, and obesity in Latin American Truck Drivers. We searched the PubMed, Web of Science, Scopus and LILACS databases, for scientific publications articles, as reported by The PRISMA Statement. From 1,382, 7 studies were included according to the established criteria. The hypertension prevalence found was 34.2%, diabetes was of 9.2% and the highest prevalence found was for overweight and obesity (56%). Meta-analysis presented that drivers have a higher prevalence of overweight or obesity when compared to eutrophic individuals and that drivers with diabetes and hyperglycemia have a lower prevalence. Due to their work activity, their access to the health system is compromised limiting any type of monitoring of their health. This study showed that there is, in Latin America, an investment and assistance gap, both in the health sector and in the research section, for this professional category, which is so important to the economy of these countries. These data should help to identify the difficulties faced by this professional in health assistance, road safety, public safety, leisure and social life. This research also highlighted that they are young and already have the first sign of non-transmissible chronic diseases, which is overweight and obesity.

**Supplementary Information:**

The online version contains supplementary material available at 10.1186/s12889-022-14902-2.

## Introduction

Heavy truck drivers represent a social group of great importance to any country's economy. In 2018, Brazil went through a truck drivers' strike that brought disruptions in the most diverse economic sectors, leaving a large part of the population with a lack of personal materials, food, and inputs, which legitimizes this professional category as fundamental for the development of the country [1].

According to a survey carried out by the National Transport Confederation (CNT), Brazil has 2 million active truck drivers, in the young adult age group with 18 years of experience in the activity [2]. These are the professionals responsible for supplying around 70% or more of cargo and goods in the Brazilian national territory.

The truck driver's professional activity requires a high level of dedication. They commonly work more than 10 h daily to meet goals [3, 4]. Due to the irregular hours in their work routine, the adopted habits mostly predispose them to a diversity of health problems, such as diabetes, hypertension, and obesity,[5] placing truck drivers in a situation of vulnerability [6].

In countries such as Japan [7], the United States of America [8], Germany [9], Canada [10], and Australia [11], there has been a concern to lifestyle, health and disease risk factors experienced among drivers, therefore investigations of the vulnerability of the truck driver about chronic diseases have been done.

In Latin America, several studies were found involving this population concerning various risk factors. Pereira et al [12] investigated the association between risk factors for the health of truck drivers and previous use of illicit drugs, in Brazil. Yonamine et al [13] investigated alcohol consumption and the use of stimulant drugs in Brazilian truck drivers. Sinagawa et al [14] reported the use of the stimulants amphetamines and cocaine by truck drivers in Brazil. However, investigations focusing on the prevalence of chronic diseases in this population are scarce. In addition, to date, no research that identifies the prevalence of diabetes mellitus, arterial hypertension, and obesity in truck drivers throughout Latin America were done. Therefore, this systematic review aimed to search the literature for information on the prevalence of chronic diseases in truck drivers in Latin America.

## Methodology

### Research and literature search strategy

The research was performed according to the Preferred Reporting Items for Systematic Reviews and Meta-Analyses (PRISMA) Statement [15], and recorded on the PROSPERO database (https://www.crd.york.ac.uk/prospero/) under protocol number: CRD42022297417.

The first stage of the research consisted of an exhaustive search and definition of MeSH terms by two researchers (RBP and SVZV) that were then validated by an expert (MDBC). The descriptors were defined independently, by researchers and, always validated by consensus.

The descriptors were divided into three blocks: [1] truck drivers, [2] chronic diseases (hipertension OR diabetes OR thrombosis OR metabolic syndrome OR Non-transmissible chronic diseases) and, [3] prevalence, to ensure greater accuracy. For LILACS, the research was carried out with the same descriptors of the other databases, but in Portuguese, providing total reproducibility. The three blocks were than combined providing a large number of abstracts. The search strategy for each database is presented on supplement 1 (SUP1).

The second stage was carried out to search the literature of the following databases: Latin American and Caribbean Health Literature in Health Sciences (LILACS), PubMed, Web of Science and Scopus. The search was done from April 1, 2021, to August 30, 2021. For this, six researchers (ACJA, CP, FSO, MRB, VDM and, SVZV), entitled Group 1, screened the articles found in the databases by reading titles and abstracts.

They conducted searches independently and any divergences were solved between them and/or by the specialists (RBP and MDBC). After that, the full text of the articles were randomly distributed to researchers in Group 1 for reading and collecting data. The selected articles were distributed to three independent reviewers (RBP, MDBC and SVZV), entitle Group 2, for certification. The final selection of publications was made by mutual agreement between researchers from both groups.

### Eligibility and exclusion criteria

The inclusion criteria considered cross-sectional retrospective observational studies that described prevalence of chronic diseases (hypertension, diabetes, thrombosis and metabolic syndrome) in truck drivers in Latin America. Articles without the prevalence data or from outside Latin America were excluded. Review studies, systematic review, meta-analysis, patent, comparative study, comments, editorial, congress, integrative-comprehensive review; in vitro and in vivo studies, language (except English, Portuguese and Spanish) and no summary available were also excluded.

### Data extraction

The reviewers from Group 1, divided into three groups (CP and MRB; FSO and ACJA; VDM and SVZV) performed the data extraction independently and the disagreements were solved by specialists. General characteristics of the studies were collected, such as authors, year of publication, time when the data were collected, where the study was conducted (country, city, etc.), prevalence of chronic diseases, and main conclusions.

### Data analysis

To perform the meta-analysis, odds ratio (OR) were the estimated effect size used, pooled with a 95% confidence interval (CI) to evaluate the prevalence of overweight or obesity, diabetes or hyperglycemia and hypertension variables, among truck drivers. The measures of the estimated effect of prevalence were presented to generate the effect size to elaborate the forest plot. Fixed and random effects were used. Heterogeneity was calculated by the χ2 test considering the I^2^ index to quantify the degree of heterogeneity within the studies. The statistical heterogeneity of the two studies was performed according to Cochran’s Q statistical test (*P* < 0.05) as indicative of significance. If the heterogeneity was high (I² > 50%), the random-effects model was chosen [16]. The meta-analysis was performed using the DerSimonian-Laird random-effects model to weight each study [17]. In addition, possible publication bias was verified by both Begg’s and Egger’s tests [18, 19] with significance *P* < 0.05. All statistical analyses were performed using the Stata software version 12.0 [Stata Corporation, College Station, TX, USA].

### Risk of bias

The methodological quality assessment tools that were used were the JBI critical appraisal checklist for studies reporting prevalence data [20], and Crombie's items for assessing the quality of cross-sectional study [21]. To provide a better sense of the bibliographic search, all reference lists of the original articles that were included in the systematic review were selected manually by Group 1. This stage of the study was highly relevant because it allowed the identification of publications that were not found in the searches of the database according to predefined search strategies and descriptors.

## Results

**Fig. 1:**
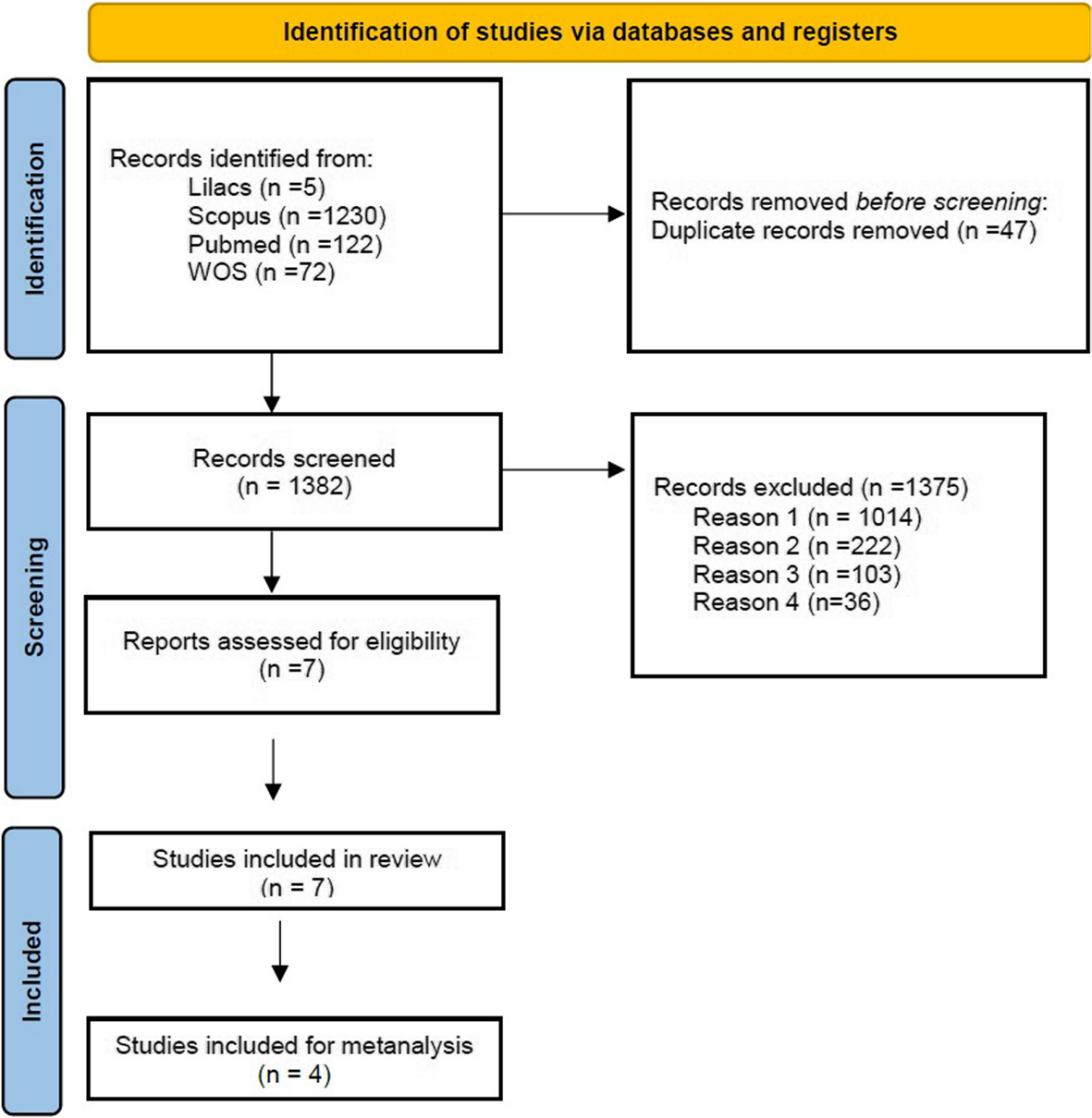
Preferred reporting items for systematic reviews and meta-analyses (PRISMA) 2020 flow diagram. Reason 1: There were no prevalence data of Hypertension or Diabetes or Metabolic syndrome or Thrombosis or Non-transmittable Chronic diseases for Truck Drivers. Reason 2: Review studies, systematic review, meta-analysis, patent, comparative study, comments, editorial, congress, integrative-comprehensive review, clinical cases, cases series, or with abstract not available. Reason 3: Study not done in Latin America´s country, city or region. Reason 4: in vivo or in vitro studies

After applying the first strategy, 1,429 studies were found, from those, 47 were excluded because of duplication. Based on the title and the abstract analysis, 1,375 articles were not included remaining 7 studies for full-manuscript analysis. All seven were used for systematic review and 4 of them to perform the meta-analysis (Fig. 1).

The main findings of selected papers are shown in Table 1. The publication period of the studies ranged from 2008 to 2020, but with data collected from 2005 to 2014. One interesting point is that all selected studies were carried out with truck drivers in Brazil. One study had the greater amount of participants, 2,228 (S1), the others ranged from 670 (S3 and S6) to 57 (S7) truck drivers.

**Table 1:** Main findings of the studies that presented prevalence of Hypertension, Diabetes and Overweigh and Obesity in Latin American Truck Drivers

**Nº**	Authors	Year of Publication	Year of data collection	Study location	Sample size	Investigated Diseases	Conclusion
S1	Mansur AP et al. [22]	2015	2006–2011	Brazilian national territory	2228	Smoking, Dyslipidemia, Diabetes, Arterial Hypertension, Sedentary Lifestyle and Obesity	Lifestyle changes and control of cardiovascular risk factors can reduce drowsiness and, therefore, decrease freight vehicle accidents
S2	Reis LAP et al [23]	2017	2014	Aparecida de Goiânia- GO	155	Obesity, Hypertension, Diabetes Mellitus	Most truck drivers had a sedentary lifestyle associated with high prevalence of overweight and obesity. High BMI was directly associated with hypertension
S3	Girotto E et al [24]	2016	2012	Paranaguá- PR	670	Chronic pain in general Arterial Hypertension Dyslipidemias, Hemorrhoids and Diabetes Mellitus	The epidemiological and pharmaco therapeutic profile of truck drivers is similar to the general population, especially regarding cardiovascular diseases and the use of medication for their treatment, with the exception of hypertension. The under treatment of identified diseases and the relationship between some professional characteristics (time of experience, employment relationship and ownership of the truck) and the use of medications stand out. Working conditions may have contributed to the increase in the prevalence of some diseases, favoring a greater continuous use of medicines
S4	Cavagioni LC & Pierin AMG [25]	2010	2005	Highway BR-116 Paulista Regis Bittencourt section—Km 312, 320 e 323	258	High blood pressure and obesity	The presence of hypertension, overweight and obesity in these professionals was expressive. Another important observation was the consumption of alcoholic beverages and the use of drugs to inhibit sleep, which could increase the rate of car accidents
S5	CavagionI, LC & Pierin, AMG [26]	2008	2005	Highway BR-116 Paulista Regis Bittencourt section—Km 312, 320 e 323	258	Metabolic Syndrome (Hypertension, Diabetes Mellitus and risk of cardiovascular disease)	The results showed a high frequency of cardiovascular risk factors in truck drivers, especially HA, overweight, obesity and sedentary lifestyle
S6	Girotto E et al [27]	2020	2012	Paranaguá- PR	670	Overweight or Obesity	More than half of the drivers presented health risk eating behaviors, reinforcing the need for strategies to encourage the reduction of these habits
S7	Marqueze EC et al [28]	2013	2009	São Paulo, SP	57	Cardiovascular diseases (Sedentary lifestyle, Obesity, Hyperlipidemia, Arterial Hypertension, Diabetes Mellitus, Metabolic Syndrome)	Truck drivers are exposed to cardiovascular risk factors due to the characteristics of their work, with high demand, extensive working hours and working time in the profession, regardless of work shift and leisure-time physical activity

**Fig. 2:**
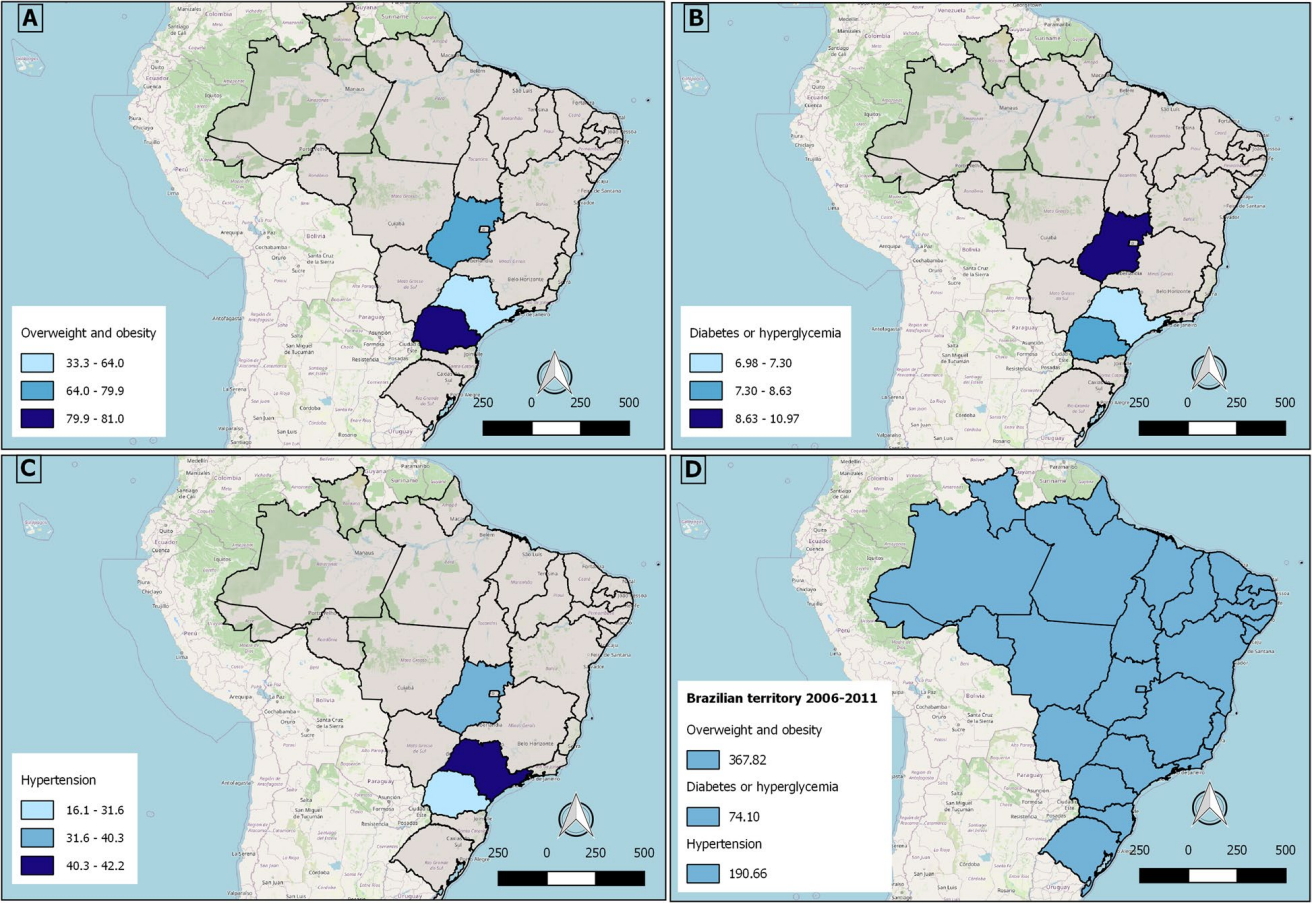
Distribution of the prevalence of Hypertension or Diabetes or Overweight and Obesity in Latin American Truck Drivers identified by the 7 selected studies in this review. **A** Prevalence of Overweight or Obesity; (**B**) prevalence of Diabetes or Hyperglycemia; (**C**) prevalence of hypertension. Mansur et al. (**D**), brought a larger sample, carried out in several points of Brazilian Highways, throughout the national territory

It is noteworthy that there are no studies on the prevalence of diabetes mellitus, hypertension and obesity in truck drivers, carried out in other Latin American countries; all of them are in Brazil, as shown in Fig. 2. The S1 study—Mansur et al [22], brought a larger sample, carried out in several points of Brazilian Highways, throughout the national territory. Thus, it was not possible to identify this study at the map (Fig. 2).

To ensure the quality of the studies that participated in the Systematic Review, an Assessment of the methodological quality (Risk of Bias) of the studies was carried out using two methods: Crombie's items (Table 2) and Joanna Briggs Institute—JBI (Table 3). Of the seven articles, both in the Crombie's method and in the JBI, two studies did not fully meet the evaluated items, but they are within the acceptable quality standard, which does not compromise their importance to participate in the present research. Therefore, they remained in this Systematic Review.

**Table 2:** Evaluation of the selected studies quality using Crombie´s items for assessing the quality of cross sectional studies

	**S1**	**S2**	**S3**	**S4**	**S5**	**S6**	**S7**
1. *Appropriateness of design to meet the aims*	Yes	Yes	Yes	Yes	Yes	Yes	Yes
2. *Adequate description of the data*	Yes	Yes	Yes	Yes	Yes	Yes	Yes
3. *Report the response rates*	Yes	Yes	Yes	Yes	Yes	Yes	Yes
4. *Adequate representativeness of the sample to total*	Unclear	Unclear	Yes	Yes	Yes	Yes	Yes
5. *Clearly stated aims and likelihood of reliable and valid measurements*	Yes	Yes	Yes	Yes	Yes	Yes	Yes
6. *Assessment of statistical significance*	Yes	Yes	Yes	Yes	Yes	Yes	Yes
*7. Adequate description of statistical methods*	Yes	Unclear	Yes	Yes	Yes	Yes	Yes
Total	6.5	6.0	7.0	7.0	7.0	7.0	7.0

Response options: Yes (1 point), Unclear (0.5 point), No (0 point). S1 Mansur AP et al., S2 Reis LAP et al., S3 Girotto E et al., S4 CavagionI LC & Pierin AMG., S5 Cavagioni, LC & Pierin, AMG., S6 Girotto E et al., S7 Marqueze EC et al

**Table 3:** Evaluation of the selected studies quality using JBI Critical Appraisal Checklist for Studies Reporting Prevalence Data

	**S1**	**S2**	**S3**	**S4**	**S5**	**S6**	**S7**
1. Was the sample frame appropriate to address the target population?	Unclear	Unclear	Yes	Yes	Yes	Yes	Yes
2. Were study participants sampled in an appropriate way?	Unclear	Unclear	Yes	Yes	Yes	Yes	Yes
3. Was the sample size adequate?	Unclear	Unclear	Yes	Yes	Yes	Yes	Yes
4. Were the study subjects and the setting described in detail?	Yes	Yes	Yes	Yes	Yes	Yes	Yes
5. Was the data analysis conducted with sufficient coverage of the identified sample?	Yes	Yes	Yes	Yes	Yes	Yes	Yes
6. Were valid methods used for the identification of the condition?	Yes	Yes	Yes	Yes	Yes	Yes	Yes
7. Was the condition measured in a standard, reliable way for all participants?	Unclear	Yes	Yes	Yes	Yes	Yes	Yes
8. Was there appropriate statistical analysis?	Yes	Unclear	Yes	Yes	Yes	Yes	Yes
9. Was the response rate adequate, and if not, was the low response rate managed appropriately?	Yes	Yes	Yes	Yes	Yes	Yes	Yes
Overall appraisal: Include □ Exclude □ Seek further info □	Include	Include	Include	Include	Include	Include	Include

Answers: Yes, No, Unclear or Not/Applicable. S1 Mansur AP et al., S2 Reis LAP et al., S3 Girotto E et al., S4 CavagionI LC & Pierin AMG., S5 Cavagioni, LC & Pierin, AMG., S6 Girotto E et al., S7 Marqueze EC et al

**Fig. 3:**
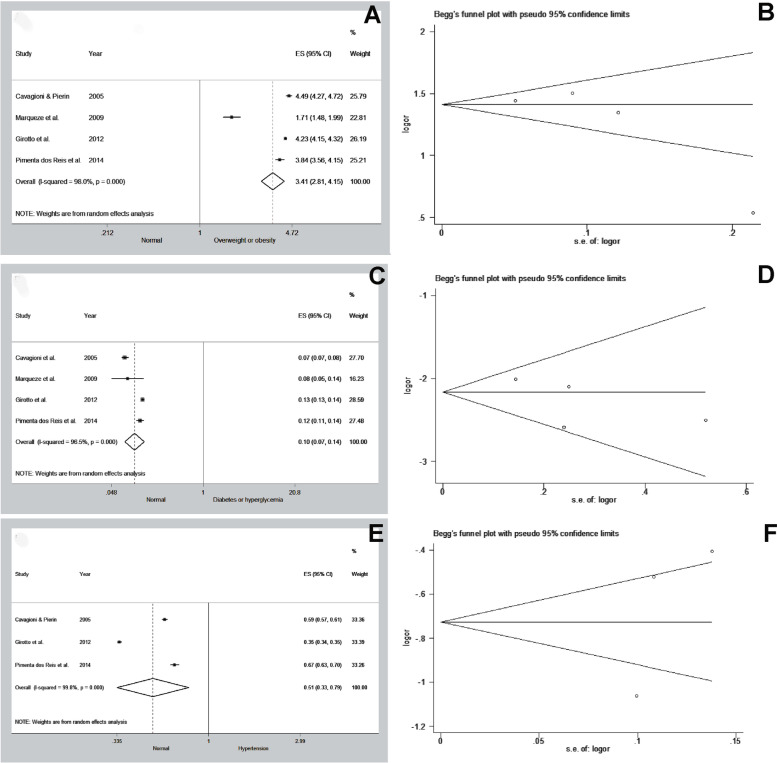
Forest plot of the prevalence rate of Overweight and obesity in 4 epidemiological studies (**A**), Diabetes or hyperglycemia in 4 epidemiological studies (**C**) or Hypertension (**E**) in 3 epidemiological studies according to random effects analysis (ES, effect size estimate); IC, interval confidence. Begg’s funnel plot for assessing publication bias in meta-analysis of Overweight and obesity in 4 epidemiological studies (**B**), Diabetes or hyperglycemia in 4 epidemiological studies (**D**) or Hypertension (**F**) in 3 epidemiological studies developed in Brazil. The funnel graph shows the effect measure (*Odds Ratio*) and standard error (S.E.) for each study

On Table 4, the main results of the meta-analysis of the variables overweight or obesity, diabetes or hyperglycemia and hypertension are presented. The variable overweight or obesity shows that drivers have a higher prevalence of overweight or obesity when compared to eutrophic individuals (OR: 3.413; 95% CI: 2.808 to 4.148; *p* < 0.001), as shown in Fig. 3A. The risk of bias assessment presents the funnel plot with slight asymmetry, indicating a possible risk of bias, however, the Egger test (*p* = 0.230) was not significant (Fig. 3B; Table 4). The forest plot of variable diabetes or hyperglycemia (Fig. 3C) demonstrates that when compared to individuals without diabetes or hyperglycemia, the drivers with diabetes and hyperglycemia have a lower prevalence (OR:0.103; 95% CI:0.075–0.142; *p* < 0.001). The funnel plot and Egger's test (*p* = 0.372) show a low risk of bias (Fig. 3D; Table 4). And the forest plot in Fig. 3E shows that drivers with hypertension have a lower prevalence when compared to individuals without hypertension (OR: 0.514; 95% CI: 0.335–0.791; *p* = 0.002). However, the funnel plot (Fig. 3F) shows asymmetry, which indicates a risk of publication bias, although Egger's test is not significant (*p* = 0.435).

**Table 4:** The main metanalysis data

		**Test of association**				**Heterogeneity test**			**Publication bias (egger's test)**
	**nº of Studies**	**OR**	**95% Cl**	***p*-value**	**Model**	**X²**	***p*-value**	**I² (%)**	***p*-value**
Overweight or obesity	4	3.413	2.808–4.148	< 0.001	Random	153.52	0.000	98.0	0.230
Diabetes or hyperglycemia	4	0.103	0.075–0.142	< 0.001	Random	86.67	0.000	96.5	0.372
Hypertension	3	0.514	0.335–0.791	0.002	Random	941.31	0.000	99.8	0.435

*OR* Odds ratio, *CI* Confidence interval

## Discussion

This is the first systematic review with meta-analysis that presents the prevalence of arterial hypertension, diabetes mellitus and obesity in Latin America truck drivers. There were only a few studies, identified in our search, that investigated the prevalence of chronic diseases in this population. This scenario evidences that even though truck drivers are a profession extremely important for the economy in Latin America [29] it is an insufficiently studied group.

Prevalence studies are relevant because they reflect the importance of different diseases for the society, but one limitation is that the risk of bias assessment of this type of study is heterogeneous and often neglected [30]. To minimize this limitation we used two methodological quality assessment tools for cross-sectional studies, the Crombie's items and the JBI Critical Appraisal Checklist for Studies Reporting Prevalence Data. On both tools, the same two studies (S1 and S2) were not positively appraised in a few parameters, overall this did not influence in their methodological quality.

Latin American countries depend heavily on road transport, a. According to Barros [29], in Brazil, about 60% of the TKT (ton per useful kilometer traveled) in 2015 were transported by highways; in Colombia this figure reached 77%, and in Mexico, 90%. Unlike what happens in other countries, where, for example, in China only 21% of the TKT passes through the roads and in the United States, only 31% [29].

The US National Institute of Occupational Safety and Health published a survey on the planning and implementation of actions that promote the health of this professional category, showing that, in recent years, there has been a significant improvement in the health of truck drivers, both physiologically and in relation to mental health [31].

According to WHO [32] hypertension, or elevated blood pressure, is a serious medical condition that significantly increases the risks of heart, brain, kidney and other diseases. The prevalence of hypertension among truck drivers found through this review was 34.2%, a value greater than the estimated hypertension prevalence (24.5%) of the Brazilian adult population [33]. Other authors also observed similar prevalence data among truck drivers in low-income (31.5%) and high-income (28.5%) countries [34].

A study with Indian truck drivers observed a lower value of 24.7% [35], as well as a study carried out in Ethiopia that described a 20% prevalence of hypertension in truck drivers [36]. In the other hand, a study in the United States described a hypertension prevalence of 34.6% [37].

The number of adults (aged 30–79 years) with hypertension increased worldwide, from 594 million in 1975 to 1.13 billion in 2015. This increase, in low- and middle-income countries is calling attention; and it is due to the rise in hypertension risk factors in those populations [32].

A study published at The Lancet analyzed blood pressure measurements of more than 100 million people, taken over three decades, in 184 countries. Prevalence was lower in Canada and Peru and higher in western countries. The prevalence exceeded 50% for men in nine countries of Central and Eastern Europe, Central Asia, Oceania and Latin America [38].

The increase in hypertension prevalence reflects different population's living conditions aspects. Aging, added to the adoption of unhealthy lifestyles, with the prioritization of ultra-processed foods, alcohol consumption, smoking and lack of physical activities, have contributed to this increase. Other aspects such as lack of knowledge, control and treatment of hypertension are also highly sensitive to individual and socioeconomic attributes [39].

Truck driver’s population also have these risks behavior. Mansur et al [22] and Reis et al [23] highlighted that Brazilian truck drivers, have high hypertension prevalence, and have unhealthy lifestyles such as use of alcohol and tobacco, not exercise regularly and not having a healthy diet.

Diabetes is a chronic, metabolic disease characterized by elevated levels of blood glucose (or blood sugar), which leads over time to serious damage to the heart, blood vessels, eyes, kidneys and nerves [40]. In this review, it was observed that 9.2% of truck drivers had altered capillary blood sugar values.

These results are well above the estimated prevalence of diabetes in the Brazilian adult male population, which is approximately 7.4% [33] and lower when compared to the values of the research by Yosef [36] who found a glycemic rate of 8% in truck drivers in Ethiopia. In contrast to these findings, a prevalence of 21.8% of altered capillary blood glucose in Brazilian truck drivers were described [41], this may be due to a small local sample (64 professionals with a mean age of 43). The difference among the studies in this review could be attributed to young age and exposure to risk factors and unhealthy lifestyle habits.

Overweight and obesity are defined as abnormal or excessive fat accumulation that may impair health [42]. The overweight and obesity prevalence in truck drivers found through this review was the highest, 56%, which reflects that this group have a great exposure to unhealthy lifestyle habits. This condition can worsen pre-existing diseases and accelerate the occurrence of more prevalent chronic diseases in the male population. The percentage for obesity found in this study is approximately 3 times higher than the estimated (20.3%) for the general population of Brazil [33]. In Europe, the estimated prevalence is also lower, 22% in Spain [43], and 19.5% in France [44].

According to Surveillance System for Risk and Protection Factors for Chronic Diseases by Telephone Survey (VIGITEL) data, in the last 13 years there has been an increase in cases of diabetes, hypertension and obesity. The research showed that, in the period between 2006 and 2019, the prevalence of diabetes increased from 5.5% to 7.4% and high blood pressure rose from 22.6% to 24.5%. The biggest increase is in relation to obesity, which went from 11.8% in 2006 to 20.3% in 2019, an increase of 72%. [33].

Of the three investigated chronic diseases (hypertension, diabetes and obesity), what was found in the few studies carried out in Latin America, was that the prevalence that stood out in the truck driver population was of overweight and obesity. Considering the average age of 40 years, obesity is the first stage for the development of hypertension and diabetes, which demonstrates the need to take care of this population.

Considering that the truck driver is a necessary professional for the Latin American economy, the studies found are punctual and there is no program aimed at this specific population.

Brazil, the largest country in Latin America, has the Unified Health System (SUS), guaranteed by the Federal Constitution [45]. The SUS has three doctrinal principles – universality, integrality and equity, guaranteeing health for all Brazilians, regardless of any economic or social factor [46].

After 31 years of the implementation of the SUS, there are many gaps, which are still not filled; one of them is the health of the truck driver, that still do not have the doctrinal principals guaranteed [47].

The organization of the system for the care of the citizen, foresees a family health team of reference, in the region where he lives, composed at least by a doctor, nurse, nursing assistant and community health agents to provide primary care [48].

In the case of truck drivers, this type of organization cannot meet their needs. Due to their work activity, access to the system is compromised. These professionals spend little time in their homes, most of the days they are on the road. This factor is certainly limiting for any type of monitoring of their health.

## Conclusion

This study showed that there is, in Latin America, an investment and assistance gap, both in the health sector and in the research section, for this professional category, which is so important to the economy of these countries. Of the 1358 studies found and reviewed, only 7 brought the prevalence of chronic diseases, and all with small samples, specific territories, revealing a small part of the problem in Latin America.

Given the scarcity of researches about Truck drivers health in the literature, this review indicate the necessity of field and data analysis researches to identify other factors that affect the behavior of these professional and that influence their health.

These data should help to identify the difficulties faced by this professional in health assistance, road safety, public safety, leisure and social life.

It is considered that programs that support truck driver health are more likely to be successful if they are based on collected data on these various components and then designing interventions that will address them.

We hope this research stimulates a new perspective on preventing the health of truck drivers.

We think it is time to also highlight that they are young and already have the first sign of non-transmissible chronic diseases, which is overweight and obesity.

### Future perspectives

As a recommendation, considering the intricacies of their profession, and the great necessity to implement specific public policies for truck drivers we suggest 4 actions that could help guarantee a better healthcare system for this population, meeting their constitutional rights.

- 1- Definition of specific tripartite financial resources to work on Research in addition to health promotion, prevention, treatment and rehabilitation.
- 2- Ensuring access to health units services located at strategic points on federal and state highways, with alternative schedules [49].
- 3- Having an integrated information system on a national level – specific electronic medical records, which would allow for the full care and monitoring of the truck driver, regardless of their place of residence [50].
- 4- Maintaining a multiprofessional team trained to meet the specific demands of this professional category [51].

## Supplementary Information

**Additional file 1.** Supplement material 1.

## Data Availability

The datasets used and/or analyzed during the current study available from the corresponding author on reasonable request.
