# Supplementary material for: A look through Latin America truck drivers’ health, a systematic review and meta-analysis
Source: BMC Public Health. 2023 Jan 2;23:3. doi: 10.1186/s12889-022-14902-2 (PMC9809045; doi:10.1186/s12889-022-14902-2)
Supplement: Supplementary file 1 — Additional file 1. Supplement material 1. [file 12889_2022_14902_MOESM1_ESM.docx]

Supplement material 1

PubMed search criteria:

[*((((((Truck Drivers AND (ffrft[Filter])) OR (****caminho*** *AND (ffrft[Filter]))) OR (motorists AND (ffrft[Filter]))) OR (Motor Vehicle AND (ffrft[Filter]))) OR (Vehicles, Motor AND (ffrft[Filter]))) OR (Trucks AND (ffrft[Filter])) AND (ffrft[Filter])) AND ((((((((Hypertension[MeSH Terms]) ) OR (****ihipertensao****[MeSH Terms])) OR (****hypertension****[MeSH Terms]) AND (ffrft[Filter])) OR (Diabetes Mellitus[MeSH Terms] AND (ffrft[Filter]))) OR (((Thrombosis[MeSH Terms]) OR (****thrombose****[MeSH Terms])) OR (****thrombosis****[MeSH Terms]) AND (ffrft[Filter]))) OR (((****syndrome metabolic****[MeSH Terms]) OR (Metabolic Syndrome[MeSH Terms])) OR (****syndrome metabolic****[MeSH Terms]) AND (ffrft[Filter]))) OR (((****doencas nao transmissiei****[MeSH Terms]) OR (Noncommunicable Diseases[MeSH Terms])) OR (****enfermedades no trasmisibles****[MeSH Terms]) AND (ffrft[Filter])) AND (ffrft[Filter]))*](https://pubmed.ncbi.nlm.nih.gov/?term=%28%28%28%28%28%28Truck+Drivers+AND+%28ffrft%5BFilter%5D%29%29+OR+%28caminho+AND+%28ffrft%5BFilter%5D%29%29%29+OR+%28motorists+AND+%28ffrft%5BFilter%5D%29%29%29+OR+%28Motor+Vehicle+AND+%28ffrft%5BFilter%5D%29%29%29+OR+%28Vehicles%2C+Motor+AND+%28ffrft%5BFilter%5D%29%29%29+OR+%28Trucks+AND+%28ffrft%5BFilter%5D%29%29+AND+%28ffrft%5BFilter%5D%29%29+AND+%28%28%28%28%28%28%28%28Hypertension%5BMeSH+Terms%5D%29+%29+OR+%28ihipertensao%5BMeSH+Terms%5D%29%29+OR+%28hypertension%5BMeSH+Terms%5D%29+AND+%28ffrft%5BFilter%5D%29%29+OR+%28Diabetes+Mellitus%5BMeSH+Terms%5D+AND+%28ffrft%5BFilter%5D%29%29%29+OR+%28%28%28Thrombosis%5BMeSH+Terms%5D%29+OR+%28thrombose%5BMeSH+Terms%5D%29%29+OR+%28thrombosis%5BMeSH+Terms%5D%29+AND+%28ffrft%5BFilter%5D%29%29%29+OR+%28%28%28syndrome+metabolic%5BMeSH+Terms%5D%29+OR+%28Metabolic+Syndrome%5BMeSH+Terms%5D%29%29+OR+%28syndrome+metabolic%5BMeSH+Terms%5D%29+AND+%28ffrft%5BFilter%5D%29%29%29+OR+%28%28%28doencas+nao+transmissiei%5BMeSH+Terms%5D%29+OR+%28Noncommunicable+Diseases%5BMeSH+Terms%5D%29%29+OR+%28enfermedades+no+trasmisibles%5BMeSH+Terms%5D%29+AND+%28ffrft%5BFilter%5D%29%29+AND+%28ffrft%5BFilter%5D%29%29&filter=dates.2000-2021)

Scopus search criteria:

*( ( ALL ( "Truck Drivers" ) OR ALL ( "caminhão" ) OR ALL ( "motorists" ) OR ALL ( "Motor Vehicle" ) OR ALL ( "Vehicles, Motor" ) OR ALL ( "Trucks" ) ) AND ( ( ALL ( "Hypertension" ) OR ALL ( "Hipertensão" ) OR ALL ( "Hipertensión" ) ) OR ( ALL ( "Diabetes Mellitus" ) OR ALL ( "Diabetes" ) ) OR ( ALL ( "Thrombosis" ) OR ALL ( "Trombose" ) OR ALL ( "Trombosis" ) ) OR ( ALL ( "Sindrome metabólica" ) OR ALL ( "Metabolic Syndrome" ) OR ALL ( "Sindrome Metabólico" ) ) OR ( ALL ( "Doenças não transmissíveis" ) OR ALL ( "Noncommunicable Diseases" ) OR ALL ( "Enfermedades no Transmisibles" ) ) ) ) AND ( ALL ( "Prevalência" ) OR ALL ( "Prevalence" ) OR ALL ( "Prevalencia" ) ) AND ( LIMIT-TO ( SRCTYPE , "j" ) ) AND ( LIMIT-TO ( OA , "all" ) ) AND ( LIMIT-TO ( LANGUAGE , "English" ) OR LIMIT-TO ( LANGUAGE , "Portuguese" ) OR LIMIT-TO ( LANGUAGE , "Spanish" ) ) AND ( LIMIT-TO ( DOCTYPE , "ar" ) ) AND ( LIMIT-TO ( PUBYEAR , 2021 ) OR LIMIT-TO ( PUBYEAR , 2020 ) OR LIMIT-TO ( PUBYEAR , 2019 ) OR LIMIT-TO ( PUBYEAR , 2018 ) OR LIMIT-TO ( PUBYEAR , 2017 ) OR LIMIT-TO ( PUBYEAR , 2016 ) OR LIMIT-TO ( PUBYEAR , 2015 ) OR LIMIT-TO ( PUBYEAR , 2014 ) OR LIMIT-TO ( PUBYEAR , 2013 ) OR LIMIT-TO ( PUBYEAR , 2012 ) OR LIMIT-TO ( PUBYEAR , 2011 ) OR LIMIT-TO ( PUBYEAR , 2010 ) OR LIMIT-TO ( PUBYEAR , 2009 ) OR LIMIT-TO ( PUBYEAR , 2008 ) OR LIMIT-TO ( PUBYEAR , 2007 ) OR LIMIT-TO ( PUBYEAR , 2006 ) OR LIMIT-TO ( PUBYEAR , 2005 ) OR LIMIT-TO ( PUBYEAR , 2004 ) OR LIMIT-TO ( PUBYEAR , 2003 ) OR LIMIT-TO ( PUBYEAR , 2002 ) OR LIMIT-TO ( PUBYEAR , 2001 ) OR LIMIT-TO ( PUBYEAR , 2000 ) )*

Lilacs search criteria:

((truck drivers) OR (caminhão) OR (motorists) OR (motor vehicle) OR (vehicles, motor) OR (trucks)  AND ( fulltext:("1") AND db:("LILACS") AND la:("pt" OR "en" OR "es")) AND (year_cluster:[2000 TO 2021])) AND ((mh:(mh:((hypertension) OR (hipertensão) OR (hipertensión)) AND ( fulltext:("1") AND db:("LILACS") AND la:("pt" OR "en" OR "es")) AND (year_cluster:[2000 TO 2021]))) OR (mh:(mh:(diabetes) AND ( fulltext:("1") AND db:("LILACS") AND la:("pt" OR "en" OR "es")) AND (year_cluster:[2000 TO 2021]))) OR (mh:(mh:((thrombosis) OR (trombose) OR (trombosis)) AND ( fulltext:("1") AND db:("LILACS") AND la:("pt" OR "en" OR "es")) AND (year_cluster:[2000 TO 2021]))) OR ((sindrome metabólica) OR (metabolic syndrome) OR (sindrome metabólico) AND ( fulltext:("1") AND db:("LILACS") AND la:("pt" OR "en" OR "es")) AND (year_cluster:[2000 TO 2021])) OR ((doenças não transmissíveis) OR (noncommunicable diseases) OR (enfermedades no transmisibles)  AND ( fulltext:("1") AND db:("LILACS") AND la:("pt" OR "en" OR "es")) AND (year_cluster:[2000 TO 2021])))

Scielo/WOS search criteria.

(((((((TS=(Truck Drivers) OR TS=(caminhão) OR TS=(motorists) OR TS=(Motor Vehicle) OR TS=(Vehicles, Motor) OR TS=(Trucks)) AND (TS=(Hypertension) OR TS=(Hipertensão) OR TS=(Hipertensión) OR (TS=(Diabetes Mellitus)) OR ( TS=(Thrombosis) OR TS=(Trombose) OR TS=(Trombosis)) OR (TS=(Sindrome metabólica) OR TS=(Metabolic Syndrome) OR TS=(Sindrome Metabólico)) OR (TS=(Doenças não transmissíveis) OR TS=(Noncommunicable Diseases) OR TS=(Enfermedades no Transmisibles)) AND (TS=(Prevalência) OR TS=(Prevalence) OR TS=(Prevalencia))))))) *Tempo estipulado=2000-2021, Idioma da pesquisa=Inglês.*
